# Mesoporous Silica Membranes Silylated by Fluorinated and Non-Fluorinated Alkylsilanes for the Separation of Methyl Tert-Butyl Ether from Water

**DOI:** 10.3390/membranes10040070

**Published:** 2020-04-15

**Authors:** Zhaojia Wang, Liwei Hao, Feihua Yang, Qi Wei

**Affiliations:** 1 State Key Laboratory of Solid Waste Reuse for Building Materials, 69 Jinding North Road, Shijingshan District, Beijing 100041, China; 2 College of Materials Science and Engineering, Beijing University of Technology, 100 Pingleyuan, Chaoyang District, Beijing 100124, China

**Keywords:** silica membrane, fluorinated/nonfluorinated alkylsilane, silylation, post-grafting, hydrophobicity, pervaporation, methyl tert-butyl ether

## Abstract

It is of great significance to separate hazardous methyl tert-butyl ether (MTBE) from water in terms of environmental protection and human health. In the present work, α-Al_2_O_3_-suppotred silica membranes were prepared by the sol-gel and dip-coating technique. Two fluorinated alkylsilanes (1H,1H,2H,2H-perfluorooctyltriethoxysilane (PFOTES) and trifluoropropyltriethoxysilane (TFPTES)) and two non-fluorinated alkylsilanes (octyltriethoxysilane (OTES) and propyltriethoxysilane (PTES)) were adopted to silylate the silica membrane by the post-grafting method which is used for the separation of MTBE from water by pervaporation. The results show that silylation enhances the hydrophobicity of silica membranes. The silylated silica membranes are selective towards MTBE, and the MTBE/water separation factor varies with grafting agents in the order: PFOTES > TFPTES > OTES > PTES. Membranes silylated with fluorinated carbon chains seem to be more selective towards MTBE than those with non-fluorinated carbon chains. The total flux is proportional to the pore volume of silica membranes, which depends on grafting agents in the order: PTES > PFOTES > OTES > TFPTES. Considering both total flux and selectivity, the PFOTES-SiO_2_ membrane is most effective in separation, with a MTBE/water separation factor of 24.6 and a total flux of 0.35 kg m^−2^ h^−1^ under a MTBE concentration of 3.0% and a feed temperature of 30 °C.

## 1. Introduction

Posing a high risk to human health, volatile organic compounds (VOCs) are widely used in various processes such as wooden furniture manufacturing, petroleum and chemicals production, etc. [1,2,3]. The release of VOCs may lead to a serious contamination to surface/ground water, and therefore it is of great significance to separate VOCs from contaminated water [4,5]. Various techniques including condensation [6], adsorption by porous materials [7], photocatalytic degradation [8,9], catalytic oxidation [10,11], membrane separation [4,12,13], air stripping in packed columns [5] and so on have been applied for VOCs removal from air or water at laboratory or industrial scale. Recently, membrane separation has drawn a great deal of attention due to the high effectiveness of VOCs removal by membrane techniques such as pervaporation and membrane distillation [12,13,14,15,16,17,18]. Both polymer membranes [19,20,21,22] and ceramic membranes [15,16,17,18] have been intensively used. Compared to polymer membranes, ceramic membranes are more thermally stable, chemically resistant and resistant to swelling, which is ascribed to their inorganic chemical and phase composition [16].

There are various reports related to the application of ceramic membranes, i.e., TiO_2_, Al_2_O_3_, ZrO_2_, zeolite and SiO_2_ membranes in VOCs removal from water [15,16,17,18,23,24]. As a kind of common material, silica membrane has been used in gas separation [25,26], membrane reactor [27], and desalination [28] due to its intrinsic merits, for instance, ease of preparation, tunable pore size, relatively low cost and so on. Pristine silica membrane is generally believed to be hydrothermally unstable owing to the presence of hydrophilic surface hydroxyl groups, which tend to physically adsorb water. The attack of physically adsorbed water leads to further hydrolysis and condensation of silica species, thus resulting in structure deterioration [29]. Fortunately, the hydrothermal stability of silica membrane has been significantly enhanced by replacing hydroxyl groups with hydrophobic groups [26], so that hydrophobic silica membranes can be applied in an aqueous environment such as desalination [28]. To the best of our knowledge, only a few mesoporous silica membranes have been reported previously on the separation of VOCs from aqueous solution by pervaporation [30,31,32]. Pervaporation is considered as a separation process for liquid mixture not only by dense membranes, but also by porous membranes, especially microporous and mesoporous membranes [23,24,31,32]. Compared to conventional separation methods, pervaporation has numerous advantages; for instance, it can be utilized for the separation of azeotropes and close-boiling point mixtures [33]. Pervaporation is advantageous for the separation of VOCs from water due to the following features [34]: (i) low temperatures. Most pervaporation process for VOCs removal are conducted at room temperatures [15,16,18]; (ii) low pressure; (iii) high cross-flow velocities are not needed; and (iv) additional chemicals are not required.

A variety of organic groups with low surface free energy are appropriate to modify porous ceramic membranes in order to turn the materials from a hydrophilic nature into a hydrophobic one. Sadao et al. functionalized silica membranes with ethyl, n-propyl, isobutyl, phenyl, vinyl, hexyl, phenyl and vinyl groups and studied the pervaporation performance of obtained hydrophobic silica membranes [15,30,35]. Perfluoroalkylsilanes with 6–12 fluorinated carbon atoms in an alkyl chain were used to silylate TiO_2_, ZrO_2_ and Al_2_O_3_ ceramic membranes by Kujawa et al., and octyltriethoxysilane (OTES) without fluorinated carbon atoms was also chosen for comparison [12,13,16,17,18]. The authors claimed that for TiO_2_ membranes with a pore size in 2–4 nm (5 kDa), the type of silylation agents has an important impact on the effectiveness of hydrophobization, and the hydrophobic nanolayer has an influence on the VOCs’ removal performance. It is noticed that perfluoroalkylsilane with less than 6 fluorinated carbon atoms in an alkyl chain has not yet been investigated in the hydrophobization of membranes for VOCs removal previously. As reported in our previous work, adopting a hydrophobic surface modified by perfluoroalkylsilane with only one fluorinated carbon atom in an alkyl chain (trifluoropropyltriethoxysilane, TFPTES) renders superior hydrothermal stability to microporous silica membranes for gas separation [36]. However, TFPTES-silylated mesoporous silica membranes have not yet been reported for the VOCs separation from water by pervaporation.

Methyl tert-butyl ether (MTBE) is a kind of hazardous VOC and the presence of MTBE in groundwater and drinking water has become a global environmental issue [37]. In the present work, mesoporous silica membranes were deposited on porous α-Al_2_O_3_ ceramics by a sol-gel technique and dip-coating method. TFPTES (with one fluorinated carbon atom in the propyl group) and 1H,1H,2H,2H-perfluorooctyltriethoxysilane (PFOTES, with six fluorinated carbon atoms in the octyl group) were adopted to silylate mesoporous silica membranes by post-grafting approach, and propyltriethoxysilane (PTES, without fluorinated carbon atom in the propyl group) and octyltriethoxysilane (OTES, without fluorinated carbon atom in the octyl group) were used for comparison. The silylated membranes were applied to separate MTBE from water by pervaporation. We aimed to quantify the effect of silylation agents on the hydrophobicity of mesoporous silica membranes, and identify the factors that may affect the membrane performance in the MTBE/water separation. To our best knowledge, no similar work on mesoporous silica membranes has been reported previously.

## 2. Materials and Methods

### 2.1. Chemicals

Tetraethyl orthosilicate (TEOS) and methyl tert-butyl ether were purchased from Fuchen Chemical Reagent Factory (Tianjin, China). Poly (ethylene oxide)-block-poly (propylene oxide)-block-poly (ethylene oxide) (Mn = 5800, EO_20_PO_70_EO_20_, P123) and propyltriethoxysilane were obtained from Alfa Aesar (Tianjin, China). Octyltriethoxysilane, trifluoropropyltriethoxysilane, and 1H,1H,2H,2H-perfluorooctyltriethoxysilane were provided by J&K Scientific Ltd. (Beijing, China), Beijing Hwrkchemical Company Limited and Nine-Dinn Chemistry (Shanghai, China), respectively. Concentrated nitric acid (HNO_3_, 65%), absolute ethanol (EtOH, 99.98%) and acetone (AC, 99.5%) were supplied by Beijing Chemical Works. The water purification system (Ulupure, Chengdu, China) was used to produce deionized water (H_2_O) with a resistivity close to 18 Ω cm. All chemicals were of analytical grade and used without further purification unless otherwise stated.

### 2.2. Preparation of Mesoporous Silica Membranes

Porous α-Al_2_O_3_ ceramics with a diameter of 24 mm, a thickness of 2 mm and an average pore size of 32 nm were home-made by a dry-pressing and sintering method and used as supports for silica membranes. A sol-gel technique was adopted to prepare silica sol according to a TEOS:P123:EtOH:H_2_O:HNO_3_ molar ratio of 1:0.15:22:35:0.05. A certain amount of P123 was dissolved in ethanol, followed by the addition of TEOS and stirring at room temperature for 30 min. Subsequently, the mixture was added dropwise into a mixture of EtOH, H_2_O and HNO_3_. After stirring for 30 min, the mixture was transferred into a water bath and stirred at 80 °C for 4 h until the yield of clear sol. The sol was then sealed and aged in a jar.

Silica membranes were prepared by dip-coating via a dip-coater (Biolin Scientific, Gothenburg, Sweden) in a clean room (grade 100). The silica sol was first ultrasonically treated to disperse the aggregated particles and then the α-Al_2_O_3_ support was dipped into the sol for 5 s with a dipping rate of 80 mm min^−1^. The coated sample was dried at room temperature and then calcined at 400 °C for 4 h with a ramp rate of 1 °C min^−1^. In order to decrease/avoid microdefects on the surface, the coating and calcination process was repeated for four times. The residual sol was dried in a petri disk until a gel was formed, followed by calcining the gel under the same condition as the coated samples to obtain xerogel for characterization.

### 2.3. Surface Silylation

The post-grafting approach was performed at room temperature with absolute ethanol as solvent and TFPTES, PFOTES, PTES and OTES as grafting agents for surface silylation of the silica membranes. The silylation conditions, such as silylation period, silylation cycle and concentration of grafting agents, were first optimized with PFOTES as represented grafting agent as follows: (1) the silica membranes were cleaned by ethanol repeatedly to remove surface impurities and then vacuum dried at 100 °C for 24 h; (2) the dried silica membranes were immersed statically into a fixed amount of mixture with an EtOH/PFOTES molar ratio of 1:0.005 and the mixture was ultrasonically treated for different periods (12, 24, 48 and 80 h, respectively) to ensure the silica membrane surface was fully accessible to the grafting agents; (3) the silica membranes were rinsed by ethanol to remove unreacted grafting agents and dried in the atmosphere at 100 °C for 12 h; (4) the optimal silylation period was determined by measuring the water contact angle (CA) on the surface; (5) Steps (2) and (3) were repeated for once, twice and thrice except for using the optimal silylation period determined in step (4); (6) the optimal silylation cycle was determined by the water CA on the surface of silica membranes treated by step (5); (7) according to step (2) and (3), three EtOH/PFOTES molar ratios of 1:0.0005, 1:0.0.005 and 1:0.05 were chosen to determine the optimal EtOH/PFOTES molar ratio for silylation based on the optimal silylation period and cycle determined in step (4) and (6), respectively. Finally, the silica membranes were silylated with TFPTES, PTES and OTES under the above optimal conditions, respectively. The samples were denoted as xy-SiO_2_, where x referred to the grafting agent/EtOH molar ratio, namely, 0.0005, 0.005 and 0.05, respectively, and y represented the type of grafting agents, PFOTES, OTES, TFPTES and PTES, respectively.

### 2.4. Material Characterization

The morphology of silica membranes was observed by scanning electron micrograph (SEM, Helios Nanolab 600i, FEI, Hillsboro, USA). The samples were sputtered with gold to enhance electronic conductivity. The CA of water and MTBE on silica membranes was measured on the OCA 20 (Dataphysics, Filderstadt, Germany) video-based contact angle system. A water/MTBE droplet of 2 μL was injected on the membrane surface in one second. The silica xerogel powder was diluted with potassium bromide (KBr) pellets and then used for infrared spectra detection on the Nicolet 5700 (Thermo Fisher Scientific, Waltham, USA) Fourier transform infrared (FT-IR) apparatus. The pore size distribution of the α-Al_2_O_3_ support was determined by the bubble point method on the 3H-2000PB Capillary Flow Porometer (Beishide Instrument, Beijing, China). The 3H-2000TD series True Density Analyzer (Beishide Instrument, Beijing, China) was used to measure the open porosity of α-Al_2_O_3_ support. The silica xerogel powder was adopted to evaluate the pore structure of membranes, which was determined by nitrogen adsorption measurement performed at −196 °C on the ASAP 2020M (Micromeritics, Atlanta, USA) volumetric adsorption analyzer. The samples were vacuumed at 200 °C for 3 h to remove the impurities and moisture on the surface before measurement. The pore size distribution was calculated from the desorption branch of isotherm according to the Barrett–Joyner–Halenda (BJH) approach and the surface area was estimated by the Brunauer–Emmett–Teller (BET) method.

### 2.5. Pervaporation (PV)

**Figure 1:**
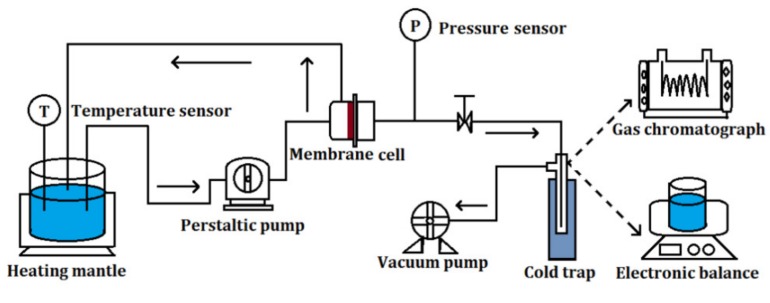
Schematic of the setup for vacuum pervaporation.

The membrane performance was evaluated with a home-made vacuum pervaporation setup as shown in Figure 1. The membrane was sealed by O-ring silicone rubber in a cylindrical stainless steel cell with a diameter of 40 mm. The flow section (effective area) of the membrane was 3.14 × 10^−4^ m^2^. A peristaltic pump was used to introduce the MTBE aqueous solution to fully contact the feed side of membrane with a flow rate of 60 mL min^−1^ or a cross-flow velocity of 3.18 × 10^−4^ m s^−1^. A vacuum pump was applied to decrease the pressure of the permeate side of the membrane to vacuum state (around 60 Pa). The permeated vapor was collected in a Dewar vessel cooling by liquid nitrogen. The mass and concentration of collected permeates were measured by electronic balance (Mettler–Toledo, Greifensee, Switzerland) and gas chromatograph (GC-2014 AT, Shimadzu, Kyoto, Japan) equipped with a thermal conductivity detector and a Porapak Q column using helium (He) as a carrier gas, respectively.

The permeate flux J and the separation factor α were calculated using the following equations:

(1) $$J=\frac{M}{A\times t}\;\left[kg\cdot m^{-2}\cdot h^{-1}\right]$$

(2) $$\alpha =\frac{y_{m}/y_{w}}{x_{m}/x_{w}}$$

where *M* refers to the total permeate weight (kg), *A* represents the effective membrane area/flow section (m^2^), *t* is the pervaporation time (h), $y_{m}\;\mathrm{and}\;y_{w}$ represent the mass percent of MTBE and water in the permeate, and $\;x_{m}\;\mathrm{and}\;x_{w}$ denote the mass fraction of MTBE and water in the feed, respectively. In the permeate mixture, MTBE may float on the water since the solubility of MTBE in water is only 4.3% (20 °C) and the density of MTBE is lower than that of water. A certain of acetone was added into the permeate mixture until a homogenous solution was obtained for gas chromatograph measurement. To evaluate the stability of membranes in the MTBE aqueous solution, the pervaporation performance was measured five times with an interval period of 10 h for each measurement.

## 3. Results and Discussion

### 3.1. Surface Silylation of Silica Membranes

**Figure 2:**
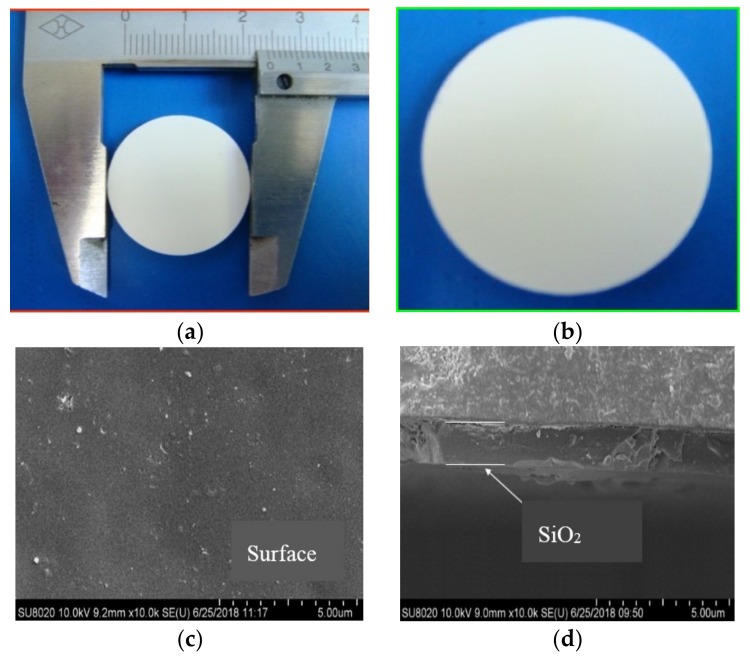
Digital photos of the samples: (**a**) α-Al_2_O_3_ support, (**b**) post-grafted silica membrane and scanning electron microscope (SEM) images of the silica membranes: (**c**) surface, (**d**) cross-section.

**Figure 3:**
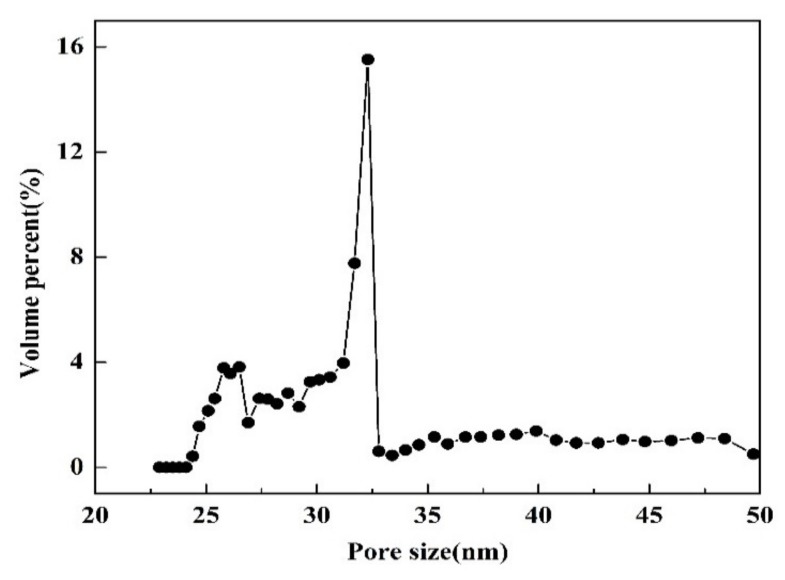
Pore size distribution of α-Al_2_O_3_ support.

Figure 2 shows the digital photos of the α-Al_2_O_3_ support (Figure 2a) and final silica membrane (Figure 2b), as well as the scanning electron microscope (SEM) images of the silica membranes (Figure 2c,d). It can be seen that the planar α-Al_2_O_3_ support has a diameter of 24 mm and a thickness of 2 mm, together with a smooth surface free of microcracks. As shown in Figure 3, the support exhibits a narrow pore size distribution centered at 32 nm. The open porosity of the α-Al_2_O_3_ support is 37.8%. A silica layer with a thickness of around 1.4 μm is successfully deposited on porous α-Al_2_O_3_ support and no obvious microdefects are present on the surface. Obviously the final grafted silica layer is transparent since the color of α-Al_2_O_3_ support remains unchanged after dip-coating with silica sol and further grafting with silylation agents. By contrast with other work [15], there is no interlayer between the support and silica layer in the present work. This is beneficial to reduce the diffusion resistance across the membrane.

**Figure 4:**
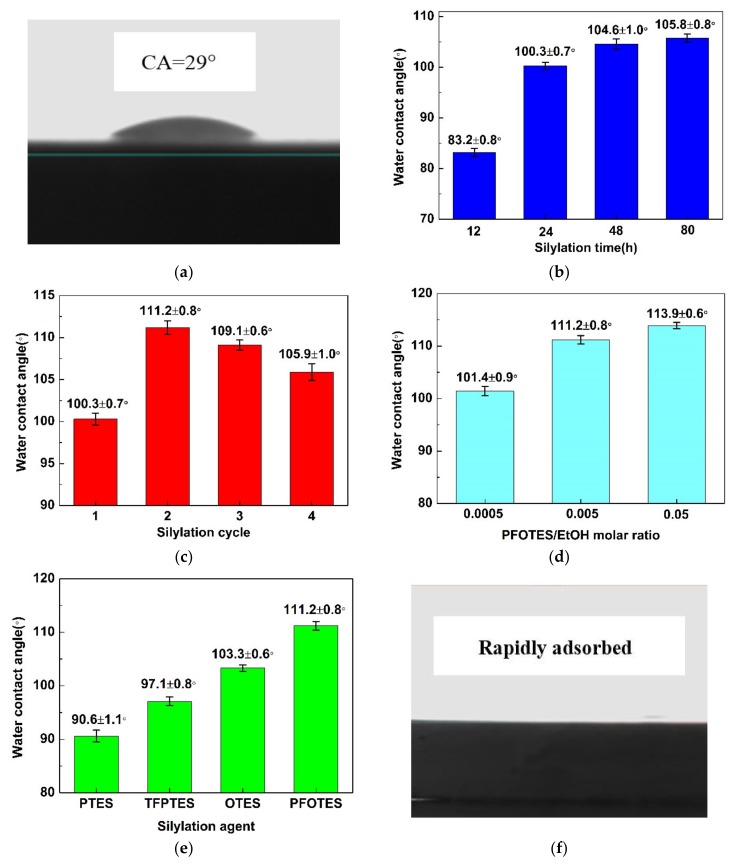
The surface silylatin of silica membranes: (**a**) water drop profile on pristine silica membrane; (**b**) silylation period dependence of the water contact angle of 1H,1H,2H,2H-perfluorooctyltriethoxysilane (PFOTES)-SiO_2_ membrane; (**c**) silylation cycle dependence of the water contact angle of PFOTES-SiO_2_ membrane; (**d**) water contact angle of silica membrane silylated with different PFOTES/EtOH molar ratio; (**e**) water contact angle of silica membrane silylated with different grafting agents; (**f**) MTBE drop profile on PFOTES-SiO_2_ membrane.

The results of surface silylation are summarized in Figure 4. It is found the water drop spreads on the surface of pristine silica membrane with a water CA of 29°, indicating that the pristine membrane is hydrophilic (Figure 4a). Figure 4b–d reveals the effect of silylation period, silylation cycle and PFOTES/EtOH molar ratio on the hydrophobicity of silica membranes. After silylation for 12 h, the silica membrane remains hydrophilic but the water CA increases to 83.2 ± 0.8°. When extending the silylation period beyond 24 h, the silylated membranes change to hydrophobic evidenced by the large water CA (>90°). The optimal silylation period is determined as 24 h, since further extending the silylation period only slightly increases the water CA from 100.3 ± 0.7° for 24 h to 104.6 ± 1.0° and 105.8 ± 0.8° for 48 and 80 h, respectively (Figure 4b). It is well-known that the hydrophobic nature originates from the condensation reaction between the surface hydroxyl groups and the ethoxy groups of grafting agents. After the reaction, the surface hydrophilic hydroxyl groups are replaced by the hydrophobic groups and hence the membranes become hydrophobic [26,34]. Meanwhile, the extension of the silylation period may generate steric hindrance, which could prevent some hydroxyl groups from being accessible to grafting agents and hinder further strengthening the hydrophobicity. Thus, multiple silylation cycles are adopted. In the second cycle of silylation, steric hindrance may be reduced by ethanol rinsing. As a result, the residual surface hydroxyl groups are able to react with the grafting agents and the water CA is increased from 100.3 ± 0.7° to 111.2 ± 0.8°. However, the hydrophobicity is weakened when further increasing the silylation cycles as the water CA decreases slightly to 109.1 ± 0.6° and 105.9 ± 1.0° upon the third and fourth silylation cycle, respectively (Figure 4c). It is possible that majority of the surface hydroxyl groups have been consumed after the second cycle of silylation, and a small quantity of grafted hydrophobic groups may be washed off upon ethanol rinsing. Thus, a cycle number of 2 is optimal for silylation. It is clear in Figure 4d that the optimal PFOTES/EtOH molar ratio for silylation is 0.005 which rendered high water CA while saving grafting agents. In a highly diluted mixture (i.e., molar ratio of 0.0005), the probability for grafting agents to react with surface hydroxyl groups decreases significantly, leading to a relatively low water CA. However, in a mixture with high PFOTES/EtOH molar ratio (i.e., molar ratio 0.05), the amount of surface hydroxyl groups is insufficient compared to the amount of grafting agents, and consequently the water CA does not increase proportionally with the concentration of grafting agents.

As shown in Figure 4e, the type of grafting agents also has an important effect on the hydrophobic property of silica membranes. The water contact angle increases with grafting agents in the order: PTES < TFPTES < OTES < PFOTES. It is noticed that for grafting agents with the same carbon chain length, for example, PFOTES vs. OTES, or PTES vs. TFPTES, the presence of C–F bonds in the chain yields a higher water CA value. This observation may be attributed to the lower surface free energy of the C–F group [38]. In the case of grafting agents with fluorinated carbon, such as PFOTES vs. TFPTES, or with non-fluorinated carbon, such as OTES vs. PTES, the longer carbon chain, the larger water CA value is obtained for silylated silica membranes. It is possible that the voids derived from the arrangement of longer carbon chains tend to trap more air than those from shorter chains, thus making the water molecules more difficult to wet the surface modified by grafting agents with long carbon chains. The wettability of MTBE on silylated silica membranes is depicted in Figure 4f. MTBE spreads rapidly on the surface and completely penetrates into the membranes, yielding a CA of 0°. This observation confirms that silylated groups are compatible with MTBE molecules. However, the silylated membranes cannot be wetted by the aqueous solution with a MTBE concentration lower than 4%.

**Figure 5:**
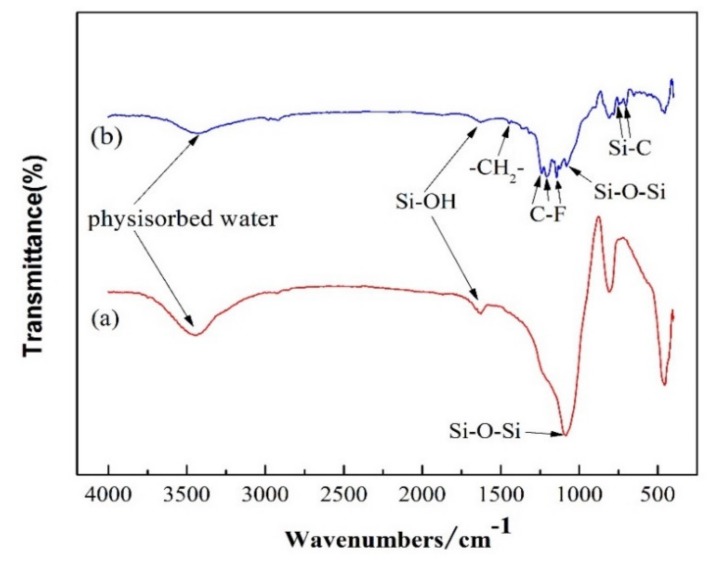
Fourier transform infrared (FT-IR) spectra of the samples: (**a**) pristine silica membrane; (**b**) PFOTES-SiO_2_ membrane.

Figure 5 shows the FT-IR spectra of pristine SiO_2_ and PFOTES-SiO_2_ membranes. The absorption peaks over the range from 1143 to 1442 cm^−1^, and 701 to 742 cm^−1^ imply the presence of the fluorocarbon groups in silylated silica membranes [39,40]. The peak at 1442 cm^−1^ corresponds to the bending vibration of –CH_2_-. The absorption at 1143, 1203 and 1239 cm^−1^ can be assigned to the stretching vibration of –CF. The absorption peaks around 701 and 742 cm^−1^ are ascribed to the Si–C bonds. However, the aforementioned absorption bands do not appear in pristine silica membranes. The signals at 1267 and 3450 cm^−1^ are attributed to residual surface Si-OH groups and the physisorbed water, respectively. The presence of the asymmetric stretching vibration of Si–O–Si at 1080 cm^−1^ indicates that the silica network is intact.

### 3.2. Pore Structure

**Figure 6:**
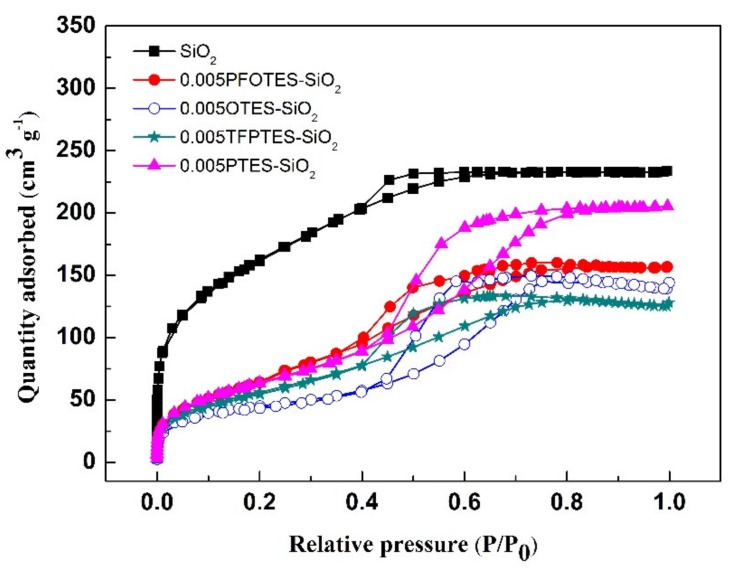
Nitrogen adsorption-desorption isotherms of pristine and silylated silica xerogels.

The pore structure of supported silica membranes is represented by the silica xerogels prepared under identical condition as silica membranes since the nitrogen adsorption measurement, a technique to detect the micro/mesoporous structure of materials, cannot be performed directly on supported silica membranes. Figure 6 shows the nitrogen adsorption-desorption isotherms of pristine and silylated silica xerogels. All samples exhibit a type IV N_2_ adsorption-desorption isotherm, suggesting the presence of a typical mesoporous structure in the samples. There is a type A hysteresis loop starting from a relative pressure of around 0.4 in the isotherms, implying that the pores have a cylindrical shape. A decrease of the amount of N_2_ adsorption is observed for all silylated samples, probably due to the blockage of pore channels by grafting agents. The reduction in N_2_ adsorption ranks in the following order for the grafting agents: PTES < PFOTES < OTES < TFPTES.

**Figure 7:**
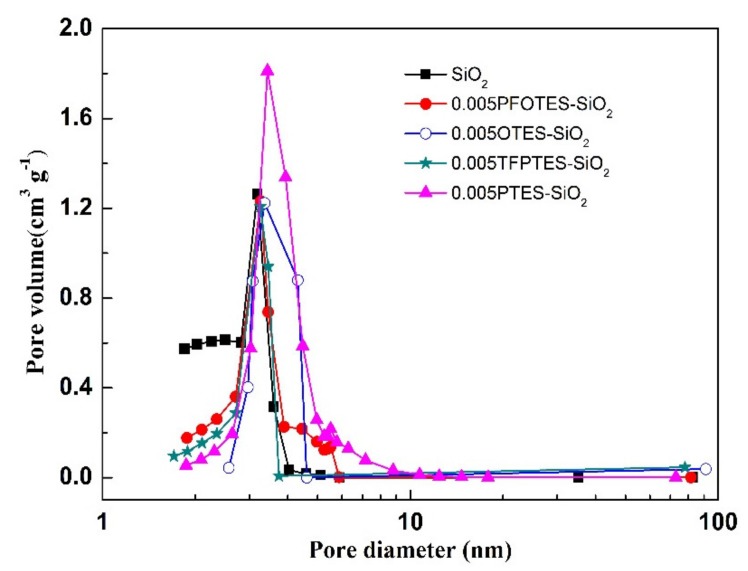
Pore size distribution of pristine and silylated silica xerogels.

The pore size distributions calculated from the desorption branch of the isotherms are displayed in Figure 7. All samples possess a narrow peak centered at a pore size ranging from 3 to 4 nm. It is noticed that the silylation does not affect the pore size dramatically.

**Figure 8:**
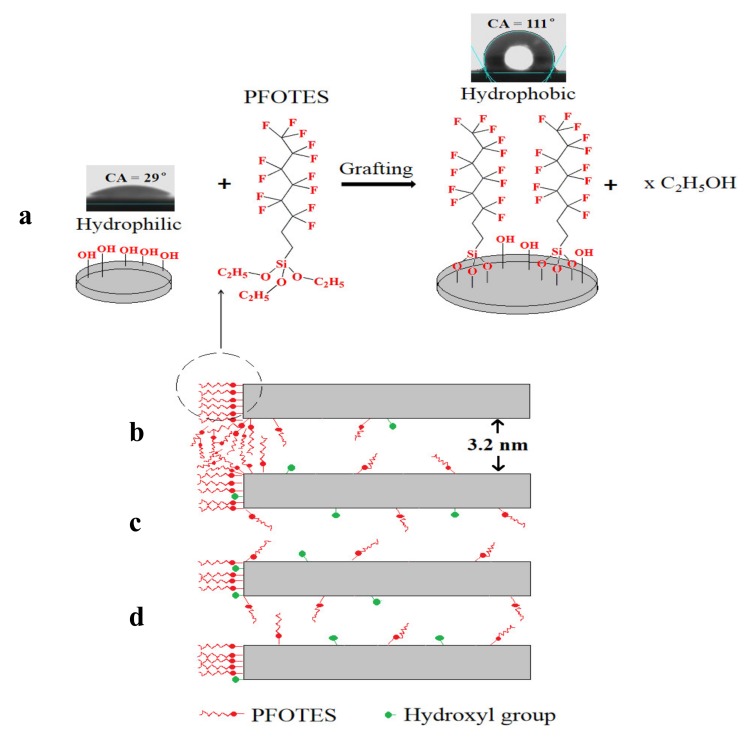
The surface silylation process and its effect on the pore structure of silica membranes. (**a**) external surface; (**b**) entrance surface; (**c**,**d**) internal surface.

The surface area, pore volume and mean pore size are summarized in Table 1. The pore volume and surface area decrease after silylation, but the pore size almost remains constant. This observation probably can be explained as follows (Figure 8). The grafting agents PFOTES, OTES, TFPTES and PTES have a molecule length of 1.44, 1.51, 0.83 and 0.96 nm, respectively, calculated on the basis of the minimum energy principle by Chem3D software. During the silylation process, the grafting agents (represented by PFOTES) preferentially react with the hydroxyl groups on external surface (Figure 8a). At the pore entrance where hydroxyl groups are abundant, the grafted molecules will extend perpendicularly from both sides of entrance surface, making the entrance narrower (Figure 8b). The aggregation of grafting agents at the narrow entrance could block pores and reduce the pore volume and surface area. It is expected that shorter molecules are more difficult to block the pore entrances, thus causing less reduction in pore volume and surface area. It can be confirmed in Table 1 that PTES molecules, which are shorter than PFOTES and OTES, lead to a less reduction in pore volume and surface area than PFOTES and OTES after silylation. However, it is surprising that TFPTES molecules have a similar length as PTES molecules, but cause the maximum decrease in pore volume and surface area after silylation among all grafting agents. Further investigation is needed to fully understand this abnormal phenomenon. Not all pore channels, especially those short of surface hydroxyl groups, are accessible to grafting agents and only a few grafting agent molecules can grow on the internal surface, and therefore these pores retain almost the same size as that of pristine membranes (Figure 8c,d).

### 3.3. Pervaporation Separation Performance of Methyl Tert-Butyl Ether (MTBE) from Water

**Figure 9:**
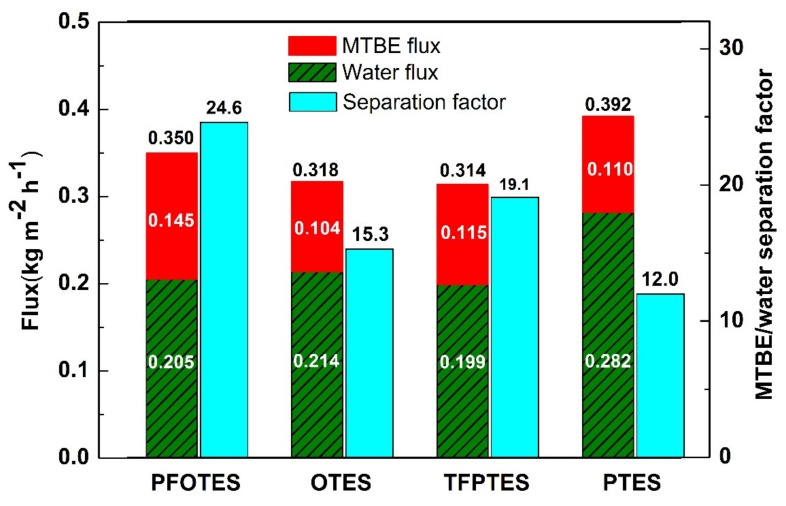
Methyl tert-butyl ether (MTBE)/water separation performance of silica membranes silylated by different grafting agents.

Figure 9 shows the flux (total, MTBE and water) and MTBE/water separation factor for the silica membranes silylated by different alkylsilanes with an alkylsilane/EtOH molar ratio of 0.005. The total flux ranges from 0.314 to 0.392 kg m^−2^ h^−1^ in the order of alkylsilane: PTES > PFOTES > OTES > TFPTES for silylated silica membranes. It is noticed that this order is the same as that of pore volume. The membrane with the highest pore volume (PTES-SiO_2_) has the largest total flux of 0.392 kg m^−2^ h^−1^, 20% higher than the lowest one (0.314 kg m^−2^ g^−1^) with the lowest pore volume (TFPTES-SiO_2_). It can be seen from Figure 9 that all silylated membranes are selective towards MTBE, and the MTBE/water separation factor varies with the type of grafting agents in the order: PFOTES > TFPTES > OTES > PTES. It is noteworthy that fluorinated carbon chains are more selective to MTBE than non-fluorinated ones. The chain length also plays a critical role in separation, for instance, the PFOTES-SiO_2_ membrane has a MTBE/water separation factor of 24.6, higher than that of the TFPTES-SiO_2_ sample (19.1) by 22%, and the separation factor of OTES-SiO_2_ is also larger than that of PTES-SiO_2_.

**Figure 10:**
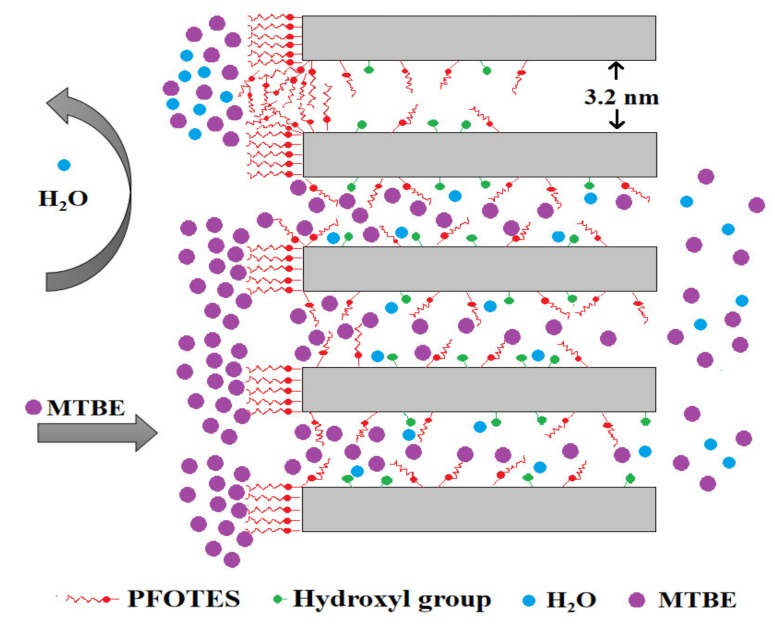
The proposed mechanism for the pervaporation separation of MTBE/water mixture by silylated silica membrane.

The pervaporation separation process complies with a solution-diffusion mechanism as described in Figure 10. It is generally believed that in pervaporation process, the separation of components depends on the difference in the rate/degree of dissolution into the membranes and the velocity of transport (also expressed as diffusivity) between the components [17,41]. In this work, MTBE and water molecules are first absorbed on the external surface of membranes at the feed side. Afterwards, the MTBE molecules dissolve selectively in the pores through moving along the surface and then diffuse across the membrane. The adsorbed water molecules also moves towards the permeate side. Finally, MTBE and water evaporate into vapor phase at the permeate side. Obviously, there is a difference in the degree of dissolution into membranes and the diffusivity across membranes between MTBE and water. It is proved that silyated groups have an affinity to MTBE molecules due to the hydrophobic interaction, so that MTBE molecules are preferentially absorbed on the silylated surface. The affinity to MTBE varies with the type of grafting groups, and the fluorinated carbon chains are more compatible to MTBE than the non-fluorinated carbon chains due to the lower surface free energy of C–F groups [38]. Owing to the more C–F groups in PFOTES than in TFPTES, the PFOTES-SiO_2_ membrane shows a higher MTBE flux and a larger MTBE/water separation factor than TFPTES-SiO_2_ membrane (Figure 9). A majority of liquid water is rejected by silylated membranes owing to the hydrophobic nature, but it is possible that some water molecules adsorb on the membranes’ surface via residual hydroxyl groups, whose presence on silylated membranes is confirmed by the FT-IR spectra as shown in Figure 5. The water flux depends on both pore volume and hydrophobicity (water CA). For instance, the highest flux occurs in the PTES-SiO_2_ membrane with the maximum pore volume and the minimum water CA. Additionally, the OTES-SiO_2_ membrane has a higher water flux than the PFOTES-SiO_2_ membrane although the OTES-SiO_2_ membrane possesses a slightly lower pore volume, and this observation is probably attributed to the larger water CA of PFOTES-SiO_2_ membrane. Apparently, the pore channels act as a pathway for the transport of MTBE and water molecules across membranes, so that the total flux increases with increasing pore volume (Figure 9). However, the selectivity is dependent on the affinity of grafting groups to MTBE.

**Figure 11:**
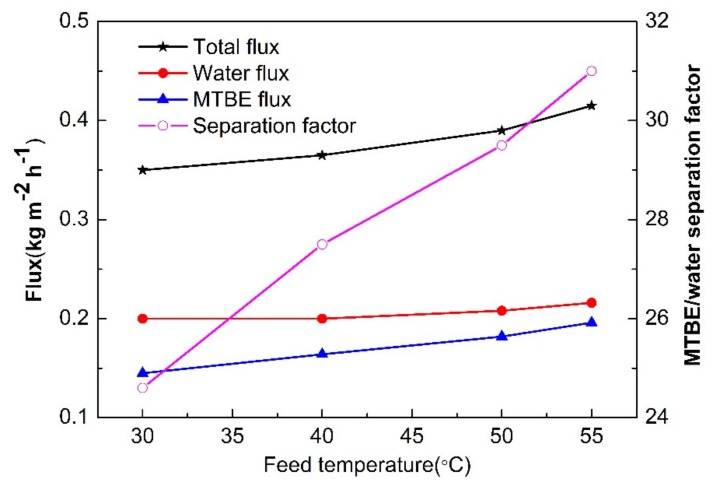
The feed temperature dependence of MTBE/water separation performance.

Figure 11 shows the effect of feed temperatures on the membrane performance. As shown, all fluxes (MTBE, water and total) increase gradually with increasing feed temperatures, but the increase of MTBE flux is more notably than that of water flux, thus increasing MTBE/water separation factor from 24.6 at 30 °C to 31 at 55 °C. With the rise of feed temperatures, the feed liquid is inclined to evaporate, resulting in an increase of vapor pressure at the feed side, and then the transport of the molecules across membranes can be additionally drived by the partial vapor pressure gradient between the feed and permeate side. At the temperature ranging from 30 to 55 °C, MTBE (with a boiling point of 55.2 °C) evaporates more intensely and, therefore, generates stronger driving force than water, thus leading to an increase in the MTBE/water selectivity. However, the selectivity is enhanced at the expense of more energy consumption and, therefore, the balance between performance and cost should be taken into account. It is noticed that the increase of flux with temperature is limited in the temperature range studied (only increasing by 18.6% from 30 to 55 °C for the total flux). This phenomenon may be related to the pore structure of silica membranes. As discussed above, the pore channels may act as pathways for mass transport. Although evaporation enhances the driving force at higher temperature, no more additional pathways are available for mass transport and then the flux is restricted. Therefore, higher porosity is required for increasing flux in silica membranes. At high temperature, membrane distillation may be more effective than pervaporation since membrane distillation is governed by the liquid–vapor equilibrium.

**Figure 12:**
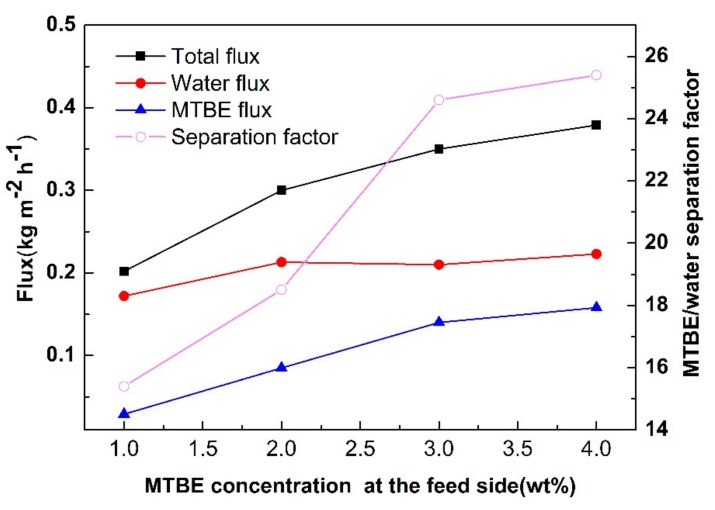
The MTBE/water separation performance varying with MTBE concentration at the feed side.

The effect of the MTBE concentration in feed solution on the membrane performance is exhibited in Figure 12. The total flux and the MTBE flux through the membranes increase obviously with increasing MTBE concentration in the feed mixture. This is due to the fact that the increasing concentration of MTBE enhances the driving force for the diffusion of MTBE through the membranes. Compared to MTBE, the water flux remains almost constant, probably due to the negligible variation in water concentration (only decreasing slightly from 99 wt% to 96 wt%). The MTBE/water separation factor increases sharply from 15.4 to 25.4 as the MTBE concentration increases from 1% to 4%.

**Figure 13:**
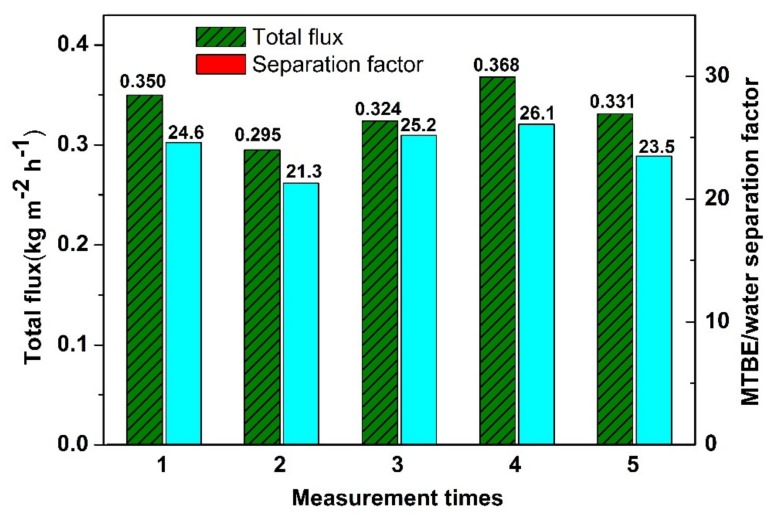
The pervaporation performance of PFOTES-SiO_2_ membrane measured at different times.

The durability of the silylated silica membranes in MTBE aqueous solution was evaluated by measuring the pervaporation performance five times with an interval period of 10 h for each measurement. It can be seen from Figure 13 that both total flux and MTBE/water separation factor do not vary significantly for each measurement, revealing that the membranes are stable in aqueous solution. The grafted hydrophobic organic groups are responsible for the hydrothermal stability of the membrane since the organic groups are more resistant to the water attack than the hydroxyl groups. The stability of hydrophobic silica membranes in aqueous solution has also been proved in previous literature [28].

In Table 2, the MTBE/water separation performance in our work is compared to those of various previous work. Our results are very comparable or even favored demonstrating a combination of high total flux and separation factor. The performance is even more promising considering the facile fabrication of silica membrane.

## 4. Conclusions

Silica membranes have been successfully prepared by a sol-gel and dip-coating technique and silylated with two fluorinated and nonfluorinated alkylsilanes by post-grafting. The silylated silica membranes switch from a hydrophilic to a hydrophobic nature, with a water contact angle ranking with grafting agents: PFOTES > OTES > TFPTES > PTES. The grafting reduces pore volume and surface area, whereas the pore size remains constant. The membranes exhibit selectivity toward hazardous methyl tert-butyl ether (MTBE) when used for the separation of MTBE from water via the pervaporation process. Membranes silylated by fluorinated carbon chains seem to be more selective than those by nonfluorinated carbon chains and the MEBE/water separation factor varies with grafting agents in the order: PFOTES > TFPTES > OTES > PTES. The total flux depends on the pore volume of the membranes. Taking both the total flux and selectivity into account, the PFOTES-SiO_2_ membrane is most effective in separation, with a MTBE/water separation factor of 24.6 and a total flux of 0.35 kg m^−2^ g^−1^ h^−1^ under a MTBE concentration of 3.0% and a feed temperature of 30 °C.

## Figures and Tables

**Table 1:** Structural parameters of the pristine and silylated silica xerogels.

Samples	Surface Area (m^2^ g^−1^)	Pore Volume (cm^3^ g^−1^)	Mean Pore Size (nm)
SiO_2_	591.3	0.36	3.2
0.005PFOTES-SiO_2_	330.9	0.24	3.3
0.005OTES-SiO_2_	303.9	0.22	3.7
0.005TFPTES-SiO_2_	269.1	0.19	3.2
0.005PTES-SiO_2_	464.0	0.31	3.4

**Table 2:** Pervaporation performance of various membranes for MTBE/water separation.

Membranes	Feed Solution	Total Flux (kg m^−2^ h^−1^)	MTBE/Water Separation Factor	References
PVAc-CSP	1wt%MTBE, 20 ℃	0.31	68	[22]
PEBAX-4033	1wt%MTBE, 40 ℃	0.01	33	[42]
Al_2_O_3_	1wt%MTBE, 40 ℃	0.7	1.1	[18]
0.005PFOTES-SiO_2_	3wt%MTBE, 40 ℃	0.35	24.6	This work

## References

[1] Tong R., Zhang L., Yang X., Liu J., Zhou P., Li J. (2019). Emission characteristics and probabilistic health risk of volatile organic compounds from solvents in wooden furniture manufacturing. J. Clean. Prod..

[2] Zheng H., Kong S., Yan Y., Chen N., Yao L., Liu X., Wu F., Cheng Y., Niu Z., Zheng S. (2020). Compositions, sources and health risks of ambient volatile organic compounds (VOCs) at a petrochemical industrial park along the Yangtze River. Sci. Total Environ..

[3] Lei Y., Ning M. (2017). Thoughts on control path of the volatile organic compounds pollution during the period of “13th Five-Year”. Sci. Environ. Prot..

[4] Altalyan H.N., Jones B., Bradd J., Nghiem L.D., Alyazichi Y.M. (2016). Removal of volatile organic compounds (VOCs) from groundwater by reverse osmosis and nanofiltration. J. Water Process. Eng..

[5] Ghoreyshi A.A., Sadeghifar H., Entezarion F. (2014). Efficiency assessment of air stripping packed towers for removal of VOCs (volatile organic compounds) from industrial and drinking waters. Energy.

[6] Khan F.I., Ghoshal A.K. (2000). Removal of volatile organic compounds from polluted air. J. Loss. Prevent. Proc..

[7] Zhang G., Liu Y., Zheng S., Zaher H. (2019). Adsorption of volatile organic compounds onto natural porous minerals. J. Hazard. Mater..

[8] Zou W., Gao B., Sik O.Y., Dong L. (2019). Integrated adsorption and photocatalytic degradation of volatile organic compounds (VOCs) using carbon-based nanocomposites: A critical review. Chemosphere.

[9] Rao Z., Xie X., Wang X., Mahmood A., Tong S., Ge M., Sun J. (2019). Defect chemistry of Er^3+^-doped TiO_2_ and its photocatalytic activity for the degradation of flowing gas-phase VOCs. J. Phys. Chem..

[10] Yang C., Miao G., Pi Y., Xia Q., Wu J., Li Z., Xiao J. (2019). Abatement of various types of VOCs by adsorption/catalytic oxidation: A review. Chem. Eng. J..

[11] He C., Cheng J., Zhang X., Douthwaite M., Pattisson S., Hao Z. (2019). Recent advances in the catalytic oxidation of volatile organic compounds: A review based on pollutant sorts and sources. Chem. Rev..

[12] Kujawa J., Al-Gharabli S., Kujawski W., Knozowska K. (2017). Molecular grafting of fluorinated and nonflfluorinated alkylsiloxanes on various ceramic membrane surfaces for the removal of volatile organic compounds applying vacuum membrane distillation. ACS Appl. Mater. Inter..

[13] Kujawa J., Kujawski W., Cyganiuk A., Dumée L.F., Al-Gharabli S. (2019). Upgrading of zirconia membrane performance in removal of hazardous VOCs from water by surface functionalization. Chem. Eng. J..

[14] He K., Wei Q., Wang Y., Wang S., Cui S., Li Q., Nie Z. (2019). Hydrophobic mesoporous organosilica membranes: Preparation and application in the separation of volatile organic compounds from water. Microporous Mesoporous Mater..

[15] Araki S., Gondo D., Imasaka S., Yamamoto H. (2016). Permeation properties of organic compounds from aqueous solutions through hydrophobic silica membranes with different functional groups by pervaporation. J. Membr. Sci..

[16] Kujawa J., Cerneaux S., Kujawski W. (2015). Removal of hazardous volatile organic compounds from water by vacuum pervaporation with hydrophobic ceramic membranes. J. Membr. Sci..

[17] Kujawski W., Kujawa J., Wierzbowska E., Cerneaux S., Bryjak M., Kujawski J. (2016). Influence of hydrophobization conditions and ceramic membranes pore size on their properties in vacuum membrane distillation of water–organic solvent mixtures. J. Membr. Sci..

[18] Kujawa J., Cerneaux S., Kujawski W. (2015). Highly hydrophobic ceramic membranes applied to the removal of volatile organic compounds in pervaporation. Chem. Eng. J..

[19] Tian X., Jiang X. (2007). Poly(vinylidene fluoride- co -hexafluoropropene) (PVDF-HFP) membranes for ethyl acetate removal from water. J. Hazard. Mater..

[20] Zhu B., Tian X., Xu Y. (2005). Recovering ethyl acetate from aqueous solution using P(VDF-co-HFP) membrane based pervaporation. Desalination.

[21] Sampranpiboon P., Jiraratananon R., Uttapap D., Feng X., Huang R. (2000). Pervaporation separation of ethyl butyrate and isopropanol with polyether block amide (PEBA) membranes. J. Membr. Sci..

[22] Yoshida W., Cohen Y. (2004). Removal of methyl tert -butyl ether from water by pervaporation using ceramic-supported polymer membranes. J. Membr. Sci..

[23] Korelskiy D., Leppäjärvi T., Zhou H., Grahn M., Tanskanen J., Hedlund J. (2013). High flux MFI membranes for pervaporation. J. Membr. Sci..

[24] Sakaki K., Habe H., Negishi H., Ikegami T. (2011). Pervaporation of aqueous dilute 1-butanol, 2-propanol, ethanol and acetone using a tubular silicalite membrane. Desalin. Water. Treat..

[25] Pelin K., Cindy H., Mieke W.J.L.-O., Arian N., Louis W. (2017). Sol-gel processed magnesium-doped silica membranes with improved H_2_/CO_2_ separation. J. Membr.Sci..

[26] Qi W., Yuan-Li D., Zuo-Ren N., Xiang-Ge L., Qun-Yan L. (2014). Wettability, pore structure and performance of perfluorodecyl-modified silica membranes. J. Membr. Sci..

[27] Scott B., Simon S., Bradley L., Shaomin L., Mikel C.D., Victor R., João CDiniz da C. (2009). Hydrothermal stability of cobalt silica membranes in a water gas shift membrane reactor. Sep. Purif. Technol..

[28] Muthia E., Christelle Y., David K.W., Simon S., João CDiniz da C. (2012). Microporous Silica Based Membranes for Desalination. Water.

[29] Duke M.C., João CDiniz da C., Do D.D., Gray P.G., Lu G.Q. (2006). Hydrothermally robust molecular sieve silica for wet gas separation. Adv. Funct. Mater..

[30] Araki S., Okabe A., Ogawa A., Gondo D., Imasaka S., Hasegawa Y., Sato K., Li K., Yamamoto H. (2018). Preparation and pervaporation performance of vinyl-functionalized silica membranes. J. Membr. Sci..

[31] Park D.H., Nishiyama N., Egashira Y., Ueyama K. (2003). Separation of organic/water mixtures with silylated MCM-48 silica membranes. Microporous Mesoporous Mater..

[32] Kim H.J., Brunelli N.A., Brown A.J., Jang K.S., Kim W.G., Rashidi F., Johnson J.R., Koros W.J., Jones C.W., Nair S. (2014). Silylated mesoporous silica membranes on polymeric hollow fiber supports: Synthesis and permeation properties. ACS Appl. Mater. Inter..

[33] Dapeng M., Dong Y., Xingyi L., Ying X., Zhaoyou Z., Yinglong W., Jun G. (2020). Mechanism analysis, economic optimization, and environmental assessment of hybrid extractive distillation−pervaporation processes for dehydration of n-Propanol. ACS Sustain. Chem. Eng..

[34] Scott K. (1995). Separation of liquid mixtures/pervaporation. Handbook of Industrial Membranes.

[35] Araki S., Imasaka S., Tanaka S., Miyake Y. (2011). Pervaporation of organic/water mixtures with hydrophobic silica membranes functionalized by phenyl groups. J. Membr. Sci..

[36] Qi W., Fei W., Zuo-Ren N., Chun-Lin S., Yan-Li W., Qun-Yan L. (2008). Highly hydrothermally stable microporous silica membranes for hydrogen separation. J. Phys.Chem. B.

[37] Zadaka-Amir D., Nasser A., Nir S., Mishael Y.G. (2012). Removal of methyl tertiary-butyl ether(MTBE) from water by polymer-zeolite composites. Microporous Mesoporous Mater..

[38] Li Q., Zhong X. (2011). Preparation and surface properties of novel low surface free energy fluorinated silane-functional polybenzoxazine films. Langmuir.

[39] Campostrini R., Ischia M., Armelao L.J. (2004). Pyrolysis study of fluorinated sol-gel silica. J. Therm. Anal. Calorim..

[40] Colthup N.B., Daly L.H., Wiberley S.E. (1990). Introduction to Infrared and Raman Spectroscopy.

[41] The Free Dictionary by Farlex. https://encyclopedia.thefreedictionary.com/pervaporation.

[42] Kujawski W., Roszak W. (2002). Pervaporative removal of volatile organic compounds from multicomponent aqueous mixtures. Sep. Sci. Technol..

